# Mukbang/Cookbang viewing and adolescent oral symptoms: A nationally representative analysis of social patterning in South Korea

**DOI:** 10.1016/j.ssmph.2026.101959

**Published:** 2026-08-09

**Authors:** Seon-Jip Kim

**Affiliations:** Department of Dental Hygiene, College of Health Science, Dankook University, 119 Dandae-ro, Dongnam-gu, Cheonan, 31116, Republic of Korea

**Keywords:** Digital food environment, Mukbang, Adolescents, Oral symptoms, Social patterning, Mediation analysis

## Abstract

Mukbang (eating-broadcast) and Cookbang (cooking-broadcast) content, a globally distributed digital food exposure originating in South Korea, reaches adolescents through algorithmically curated platforms, yet its population-level implications for oral health remain uncharacterised. Pooled cross-sectional data from 106,005 Korean adolescents (2022 and 2025 Korea Youth Risk Behavior Web-based Surveys) were analysed using design-based methods. Viewing frequency was classified as not watching (32.2%), ≤3 times/month (30.3%), 1–4 times/week (26.4%), and ≥5 times/week (11.1%); two-thirds (67.8%) had viewed such content in the preceding 12 months. Design-based logistic regression estimated adjusted odds ratios (aORs) for two patient-reported oral symptoms, toothache and gingival pain/bleeding; natural direct and indirect effects through sugary beverage intake, late-night snacking, and fast-food intake were estimated by g-computation. Viewing frequency was associated with both symptoms in a dose-response pattern. Comparing ≥5 times/week with not watching, aORs were 1.21 (95% CI, 1.14–1.28) for toothache and 1.22 (1.15–1.30) for gingival pain/bleeding (P for trend < .001). Sugary beverage intake and late-night snacking, but not fast-food intake, carried significant indirect effects, accounting for 29.6% (22.2–42.7) and 18.0% (13.2–25.5) of the respective total effects; these proportions are a design-based decomposition of cross-sectional associations and require longitudinal confirmation. The toothache association was stronger in females and middle-school students. Given that most adolescents are exposed and one in nine views this content near-daily, even modest individual-level associations imply an appreciable population burden. Korean food-advertising law already covers this age group and these food categories, but not the platforms that carry the content.

## Highlights

• Digital food-environment exposure is associated with adolescent oral symptoms at scale.
• Mukbang/Cookbang viewing showed a dose-response association with two oral symptoms.
• Sugary beverages and late-night snacking accounted for part of the association.
• Two-thirds of adolescents view this content; one in nine views it near-daily.
• Korean food-advertising law covers these foods but not the platforms carrying them.

## 1 Introduction

Adolescents are growing up within a profoundly reshaped food environment, in which the digitalisation of food-related content has emerged alongside conventional advertising and built-environment factors as a defining feature of population exposure to food and drink. This transformation is consistent with broader scholarship identifying the food environment as a structural determinant of population dietary patterns and chronic disease (Swinburn et al., 2019), and with the commercial determinants of health framework, which highlights how industries that profit from the consumption of harmful or ultraprocessed products shape population behaviours through marketing, product design, and digital ecosystems (Maani et al., 2020). Eating-broadcast (Mukbang) and cooking-broadcast (Cookbang) videos, in which hosts consume large portions of food or prepare visually appealing meals while interacting with viewers in real time, exemplify this digitalised food environment. Originating in South Korea around 2009 (Donnar, 2017) but now globally distributed through YouTube, TikTok, and similar platforms, this content reaches adolescents through algorithmically curated recommendation systems, with near-universal smartphone ownership ensuring continuous access (Ministry of Science and ICT & National Information Society Agency, 2024). In recent nationally representative Korean surveys, approximately 70% of adolescents have viewed Mukbang or Cookbang content in the past 12 months, with weekly viewing reported by a substantial minority (Sung et al., 2024); the present data give corresponding figures of 67.8% and 37.5%. Such exposure prevalence places this digital food-media format alongside the built food environment and food marketing as a high-coverage, population-relevant exposure rather than a niche behavioural curiosity.

Concerns have grown that this form of digital food-environment exposure may shape adolescent dietary behaviours through neurobehavioural pathways. Korean nationally representative studies have linked frequent Mukbang/Cookbang viewing to unhealthy dietary behaviours (Kang et al., 2020; Sung et al., 2024), higher body mass index status (Park et al., 2024), and dietary risk behaviours independent of overall smartphone use (Kim & Oh, 2024), and a measurable subset of viewers report problematic viewing patterns consistent with behavioural addiction (Kircaburun et al., 2021). Mechanistic accounts emphasise cue-induced craving and “vicarious eating”: repeated visual exposure to palatable food activates appetitive responses analogous to direct food cues and reflects the parasocial influence of charismatic hosts on adolescent food choice (Spence et al., 2019; Strand & Gustafsson, 2020). Neuroimaging in 9- to 12-year-old children further shows that dynamic (video) food cues elicit greater reward-circuitry activation (amygdala, insula, ventral tegmental area) than static images (Yeum et al., 2023), suggesting that the immersive video format of Mukbang and Cookbang may be neurobehaviourally distinct from earlier food advertising and provides a plausible upstream mechanism for downstream population dietary patterns.

These dietary pathways are well-established population-level risk factors not only for obesity and cardiometabolic disease but also for oral diseases, particularly dental caries and gingivitis. A systematic review and meta-analysis of observational studies confirmed that higher sugar-sweetened beverage consumption is associated with significantly increased odds of dental caries (OR 1.57; 95% CI, 1.28–1.92) and dental erosion (OR 1.43) (Valenzuela et al., 2021), and cohort studies in 10- and 15-year-old populations have demonstrated dose-response relationships between sugary beverage intake and caries experience (Pitchika et al., 2020). Restricting free sugars to below 10% of total energy intake is supported by systematic review evidence as effective for caries reduction (Moynihan & Kelly, 2014) and underpins current World Health Organization guidance (World Health Organization, 2015). Oral diseases remain among the most prevalent chronic conditions worldwide, exhibit a marked social gradient, and have been explicitly identified as a global public-health challenge in need of upstream, environment-focused policy responses (Peres et al., 2019).

Despite this growing evidence base linking digital food-environment exposure to dietary behaviours and weight outcomes, its implications for oral health have not been systematically examined at the population level. Oral diseases remain among the most prevalent chronic conditions of adolescence and have substantial impact on quality of life, school attendance, and the lifetime trajectory of dental disease (Petersen, 2008). Recent national data underline the timeliness of the question: an interrupted time-series analysis of 963,644 respondents to the same national survey used here found that adolescent oral-hygiene practice declined in a sustained manner through the COVID-19 pandemic period, even as hand hygiene initially improved (Oh et al., 2023). Adolescence is also a critical period during which dietary and oral-health behaviours consolidate. Whether digital food-environment exposure is associated with the population burden of oral symptoms, and whether observed associations are consistent with pathways involving specific dietary behaviours, is therefore an important question for population health, for the regulation of digital food environments, and for the broader social-science research agenda on the commercial and structural determinants of adolescent health.

Drawing on this literature, three pre-specified hypotheses were developed. First, more frequent Mukbang and Cookbang viewing would be associated, in a dose-response manner, with higher odds of patient-reported toothache and gingival pain/bleeding (H1). Second, this association would be at least partially consistent with pathways involving greater sugary beverage intake, late-night snacking, and fast-food intake, with the sugary beverage and late-night snacking pathways expected to carry the largest indirect effects given their cariogenic potency and the impaired oral clearance accompanying bedtime sugar exposure (Moynihan & Kelly, 2014; Nishide et al., 2019; Valenzuela et al., 2021) (H2). Third, the strength of association would be socially patterned, with greater effects in subgroups characterised by heightened behavioural susceptibility to food-media influence, specifically female adolescents (Lukacs, 2011; Sung et al., 2024) and younger (middle-school) adolescents (Steinberg, 2008); effect modification by depressive symptoms and household economic status was exploratory and motivated by population-health interest in mental-health and socioeconomic axes of oral-health inequality (Peres et al., 2019) (H3).

To test these hypotheses, nationally representative cross-sectional data from the 2022 and 2025 waves of the Korea Youth Risk Behavior Web-based Survey were used to (1) estimate the dose-response association between Mukbang/Cookbang viewing frequency and patient-reported toothache and gingival pain/bleeding; (2) decompose that association into components operating through sugary beverage intake, late-night snacking, and fast-food intake; and (3) examine the social patterning of the association by sex, school level, depressive symptoms, and household economic status.

## 2 Methods

### 2.1 Study design and population

Data were drawn from the Korea Youth Risk Behavior Web-based Survey (KYRBS), an anonymous, self-administered, cross-sectional national survey conducted annually by the Korea Disease Control and Prevention Agency to monitor health-risk behaviours among Korean middle- and high-school students (Kim et al., 2016). The KYRBS employs a stratified, multistage cluster sampling design to obtain a nationally representative sample, with schools as the primary sampling unit and one class per grade randomly selected within each sampled school. It samples students in the three grades of middle school and the three grades of high school, corresponding to a nominal age range of approximately 12 to 18 years (mean 15.1 years in the present sample). Survey weights, stratification, and clustering information are released with the public-use data to permit design-based population estimates.

The present analysis used pooled data from the 2022 and 2025 waves of the KYRBS, both of which included items on Mukbang and Cookbang viewing. Of 106,020 adolescents who completed the survey, 15 with missing data on study covariates were excluded, yielding a final analytical sample of 106,005 adolescents drawn from 1594 schools within 216 strata.

### 2.2 Ethical considerations

The KYRBS protocol was approved by the Korea Disease Control and Prevention Agency. The present secondary analysis of de-identified, publicly released data was approved with an exemption from full review by an institutional review board (approval reference and institution are provided on the separate title page to preserve double-blind peer review).

### 2.3 Mukbang/cookbang viewing frequency

**Fig. 1:**
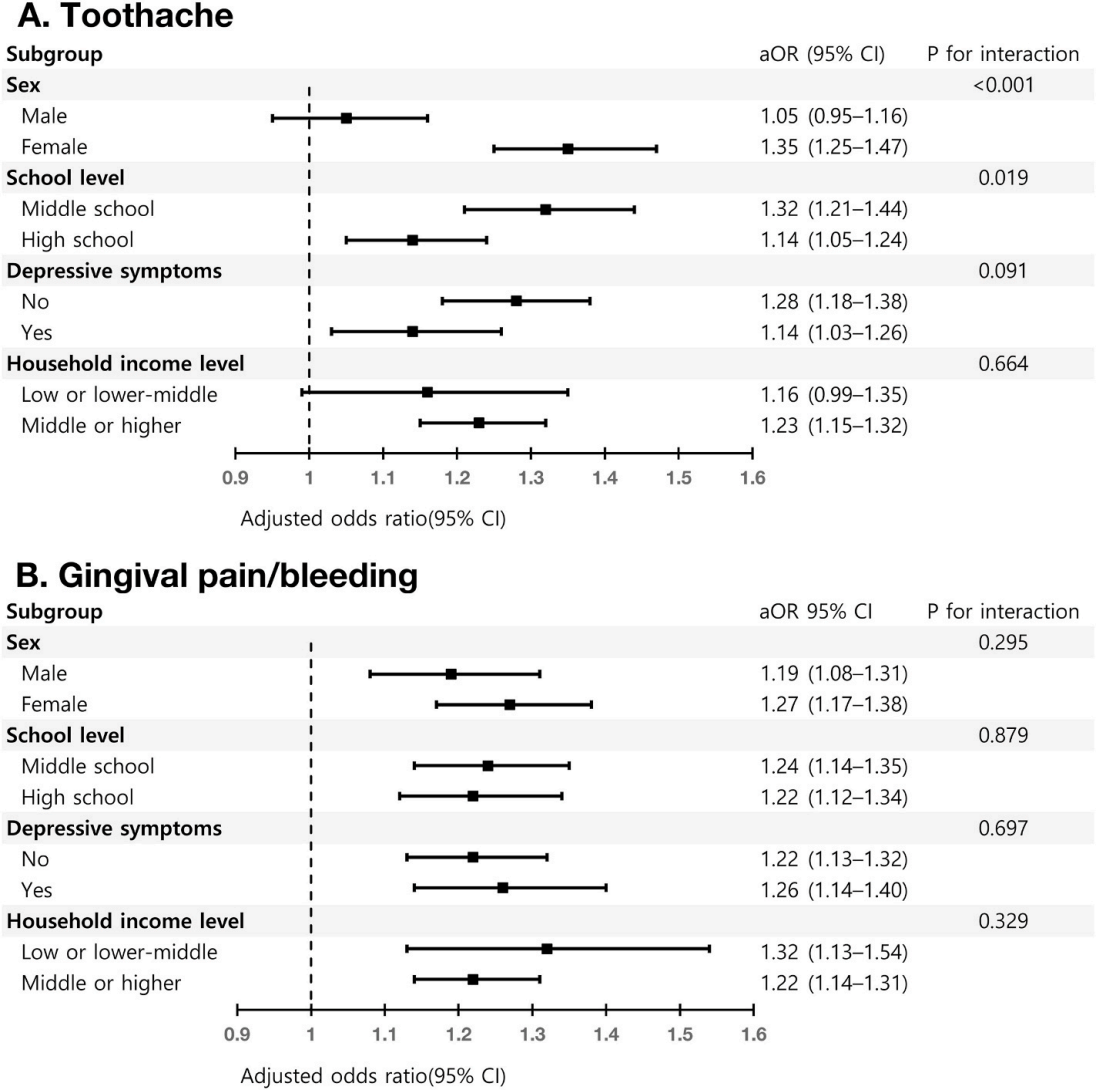
Forest plots of subgroup analyses showing adjusted odds ratios for (A) toothache and (B) gingival pain/bleeding among adolescents watching Mukbang/Cookbang ≥5 times/week versus those not watching (reference). Models within each stratum were adjusted for the covariates listed in the Methods, with the stratifying variable removed from the covariate set. P for interaction was obtained from a design-based Wald test of the product term between viewing frequency and the stratifying variable, fitted in the corresponding main-effects model.

Participants were asked how frequently they had viewed Mukbang (eating-broadcast) or Cookbang (cooking-broadcast) content during the past 12 months. The KYRBS asks a single item covering the two formats jointly; the survey does not permit them to be distinguished, and the exposure is therefore necessarily a combined measure. Responses were classified into four ordered categories: not watching; ≤3 times/month; 1–4 times/week; and ≥5 times/week. The variable was entered as a four-level categorical variable in the main dose-response analysis (Table 2), the mediation analysis (Table 3), and the subgroup analyses (Fig. 1), and as an ordinal term (0–3) in trend tests and interaction tests.

**Table 1:** Characteristics of participants according to Mukbang/Cookbang viewing frequency.

Characteristic	Overall	Not watching	≤3/month	1–4/week	≥5/week	*P*
*n* (unweighted)	106,005	33,491	32,308	28,268	11,938	
Weighted %	100.0	32.2	30.3	26.4	11.1	
**Sex**						< .001
Male	51.5	66.3	48.2	42.8	38.1	
Female	48.5	33.7	51.8	57.2	61.9	
**School level**						< .001
Middle school	51.5	50.1	54.6	50.5	49.2	
High school	48.5	49.9	45.4	49.5	50.8	
**Household economic status**						< .001
Middle or higher	89.3	89.9	89.6	89.2	87.1	
Low/lower-middle	10.7	10.1	10.4	10.8	12.9	
**Ever alcohol use**						< .001
No	69.3	72.1	71.4	66.8	61.7	
Yes	30.7	27.9	28.6	33.2	38.3	
**Ever smoking**						< .001
No	92.2	92.7	93.3	91.5	89.2	
Yes	7.8	7.3	6.7	8.5	10.8	
**Depressive symptoms**						< .001
No	72.8	76.2	73.7	70.9	64.9	
Yes	27.2	23.8	26.3	29.1	35.1	
**Toothbrushing, previous day**						< .001
0 time	1.3	1.5	1.2	1.0	1.4	
1–2 times	58.8	57.6	59.8	59.4	57.6	
≥3 times	40.0	40.8	39.0	39.6	41.0	
**Dental floss use**						< .001
No	73.9	73.4	73.4	74.6	75.4	
Yes	26.1	26.6	26.6	25.4	24.6	
**Interdental brush use**						.008
No	77.9	77.2	78.0	78.3	78.3	
Yes	22.1	22.8	22.0	21.7	21.7	
**Dietary behaviours (mediators)**, mean (SD)						
Sugary beverages, times/week	3.91 (3.73)	3.84 (3.88)	3.69 (3.35)	3.96 (3.55)	4.61 (4.49)	< .001
Late-night snacking, days/week	1.45 (1.69)	1.34 (1.72)	1.40 (1.60)	1.53 (1.66)	1.67 (1.85)	< .001
Fast food, times/week	2.01 (1.91)	1.85 (1.88)	1.95 (1.69)	2.11 (1.83)	2.39 (2.60)	< .001
**Oral symptoms (outcomes)**						
Toothache	21.0	18.4	21.1	22.4	24.8	< .001
Gingival pain/bleeding	18.1	15.8	18.4	19.4	21.1	< .001

Values are weighted percentages, except dietary behaviours, which are weighted means (SD) of approximate weekly consumption frequency (eating occasions per week for sugary beverages and fast food; days per week for late-night snacking), and the first row, which gives unweighted counts. *P* values are from Rao–Scott corrected chi-square tests (categorical variables) or design-based Wald tests (dietary behaviours). *N* = 106,005.

**Table 2:** Adjusted odds ratios (95% CI) for oral symptoms according to Mukbang/Cookbang viewing frequency.

Mukbang/Cookbang viewing frequency	Toothache, aOR (95% CI)	Gingival pain/bleeding, aOR (95% CI)
Not watching	1.00 (Reference)	1.00 (Reference)
≤3 times/month	1.12 (1.07–1.17)	1.13 (1.08–1.18)
1–4 times/week	1.14 (1.09–1.19)	1.16 (1.11–1.21)
≥5 times/week	1.21 (1.14–1.28)	1.22 (1.15–1.30)
***P* for trend**	**< .001**	**< .001**

aOR, adjusted odds ratio; CI, confidence interval. Estimates are from design-based (complex-survey) multivariable logistic regression models adjusted for sex, grade, household economic status, depressive symptoms, ever alcohol use, ever smoking, toothbrushing frequency, dental floss use, and interdental brush use. *P* for trend was obtained by entering Mukbang/Cookbang viewing frequency as an ordinal variable in the model.

**Table 3:** Design-based causal mediation analysis of Mukbang/Cookbang viewing and oral symptoms through dietary behaviours.

Effect (≥5 times/week vs not watching)	Toothache, OR (95% CI)	Gingival pain/bleeding, OR (95% CI)
Total effect	1.20 (1.14–1.27)	1.22 (1.15–1.28)
Natural direct effect	1.14 (1.08–1.20)	1.17 (1.11–1.24)
**Natural indirect effect (all mediators)**	**1.056 (1.049–1.064)**	**1.036 (1.030–1.042)**
Via sugary beverage consumption	1.030 (1.025–1.035)	1.019 (1.015–1.024)
Via late-night snacking	1.022 (1.018–1.026)	1.015 (1.012–1.019)
Via fast-food consumption	1.004 (.999–1.008)	1.001 (.996–1.005)
**Proportion mediated, % (95% CI)**	**29.6 (22.2–42.7)**	**18.0 (13.2–25.5)**

OR, odds ratio; CI, confidence interval. Effects were estimated by design-based g-computation and adjusted for the same covariates as Table 2 (see Methods). Mediator-specific effects are interventional indirect effects. Proportion mediated = log(indirect-effect OR) ÷ log(total-effect OR) × 100. CIs are percentile intervals from 2000 bootstrap resamples of primary sampling units within strata. These quantities are a design-based decomposition of a cross-sectional association and require longitudinal confirmation.

### 2.4 Oral symptoms

Two binary outcomes captured patient-reported oral symptoms in the preceding 12 months: (1) toothache, defined as having experienced any aching or throbbing tooth pain; and (2) gingival pain/bleeding, defined as having experienced any gum pain or bleeding. Each outcome was coded as present versus absent. These two items are referred to throughout as patient-reported oral symptoms, while “oral disease” is reserved for the clinically diagnosed caries and periodontal disease of the cited literature.

### 2.5 Dietary behaviours (mediators)

Three dietary behaviours were specified a priori as mediators on the basis of established cariogenic and gingival risk pathways. Sugary beverage consumption was measured by a single item asking how often the respondent drank sweetened beverages during the preceding 7 days, with seven ordered categories (did not drink; 1–2, 3–4, or 5–6 times per week; once, twice, or three or more times daily). Fast-food consumption was measured by an identically structured item. Late-night snacking was measured by an item asking on how many days in the preceding 7 the respondent ate a late-night snack, with eight ordered categories (none through 7 days).

The three items therefore differ in metric: two count eating occasions and one counts days, and the occasion-based categories are markedly non-linear in their upper range. The ordered category scores were entered as continuous variables in the primary analysis; because this treats category intervals as equal, the mediation analysis was repeated with the mediators rescaled to their approximate weekly frequencies (category midpoints) and again after logarithmic transformation (Supplementary Table S2 and S3). All three items capture frequency rather than absolute amount, which under non-differential measurement error will bias indirect effects towards the null and render the estimates conservative.

The recall windows of the three constructs are not aligned. The dietary mediators refer to the 7 days preceding the survey, whereas Mukbang/Cookbang viewing and the oral symptoms both refer to the preceding 12 months; the mediator measurement windows therefore fall within, rather than between, those of the exposure and the outcomes.

### 2.6 Covariates

Covariates were selected a priori on the basis of previous literature on adolescent oral health: sex (male, female); grade (six categories from middle-school grade 1 to high-school grade 3, included as five indicator variables with middle-school grade 1 as the reference); self-reported household economic status (five categories); depressive symptoms (felt so sad or hopeless almost every day for ≥2 consecutive weeks during the past 12 months that they stopped doing some usual activities); ever alcohol use; ever smoking; toothbrushing frequency on the previous day (0, 1–2, ≥3 times); dental floss use; and interdental brush use.

For sensitivity analyses, six further covariates were derived: average daily smartphone use time (a weighted average of separately reported weekday and weekend use); sleep duration (derived from reported bedtime and waking time); sedentary time (a weighted average of weekday and weekend sitting time); perceived stress; use of dental services in the preceding 12 months; and self-reported academic performance.

### 2.7 Statistical analysis

All analyses incorporated the complex sampling design of the KYRBS, including stratification, clustering, and survey weights, with sampling design degrees of freedom of 1378. Sample characteristics across viewing-frequency groups were compared using Rao–Scott second-order corrected chi-square tests for categorical variables (Rao & Scott, 1984) and design-based Wald tests for the dietary behaviours (Table 1).

To estimate the total effect of Mukbang/Cookbang viewing frequency on each oral symptom, design-based multivariable logistic regression models adjusted for all covariates listed above were fitted (Table 2). Adjusted odds ratios (aORs) and 95% confidence intervals (CIs) were calculated using “not watching” as the reference category, with variances obtained by Taylor linearisation. Linear trend was tested by entering viewing frequency as an ordinal term.

To decompose the total effect, natural direct and natural indirect effects operating jointly through the three dietary behaviours were estimated within the counterfactual framework by g-computation (VanderWeele & Vansteelandt, 2010; VanderWeele, 2015). Design-weighted linear models were fitted for each mediator and a design-weighted logistic model for each outcome; counterfactual means were obtained by integrating over the joint mediator residual distribution using 20-point Gauss–Hermite quadrature. This procedure does not require the rare-outcome approximation on which product-of-coefficients estimators depend, and which the prevalence of the present outcomes (Table 1) does not satisfy. The headline contrast compares viewing ≥5 times/week with not watching. Mediator-specific contributions are reported as interventional indirect effects, in which the mediator concerned is set to its distribution under frequent viewing while the remaining mediators are held at their distribution under non-viewing. The proportion mediated was calculated as log (natural indirect effect OR) ÷ log (total effect OR) × 100. Ninety-five per cent CIs are percentile intervals from 2000 nonparametric bootstrap resamples of primary sampling units (schools) drawn with replacement within strata, so that the resampling scheme reproduces the survey design.

Because the data are cross-sectional, these quantities are reported as a design-based decomposition of observed associations. The estimands take their formal names from the counterfactual framework, but their causal interpretation rests on identification assumptions that the present design cannot support (Section 4.3).

Pre-specified subgroup analyses examined whether the association between Mukbang/Cookbang viewing (≥5 times/week vs not watching) and each oral symptom varied by sex, school level (middle vs high school), depressive symptoms, and household economic status (Fig. 1). Effect modification was assessed by adding a product term between viewing frequency and the stratifying variable to the main-effects model and testing it with a design-based Wald test referred to the F distribution on the sampling design degrees of freedom. A likelihood ratio test is not available under pseudo-likelihood estimation for complex survey data and was not used. Stratum-specific estimates were obtained by refitting the model within each stratum, with the stratifying variable removed from the covariate set. In a further analysis, age was entered as a continuous variable (12–18 years, centred at 15) in place of the grade indicators, with a product term with ordinal viewing frequency (Supplementary Table S4).

Sensitivity analyses successively adjusted the main models for smartphone use time, sleep duration, sedentary time, perceived stress, dental-service use, and academic performance, and repeated the mediation analysis under the extended covariate set, under a model allowing exposure–mediator interaction, and under two alternative mediator scalings (Supplementary Table S1–S3).

Analyses were conducted in Python 3.12.3 (NumPy 2.4.4, SciPy 1.17.1, pandas 3.0.2, pyreadstat 1.3.5) using purpose-written design-based routines. As a check on the implementation, the design-based logistic regression reproduced the SPSS Complex Samples (CSLOGISTIC) estimates for Table 2 to three decimal places, and the Rao–Scott tests reproduced the corresponding CSTABULATE output. Analysis code is provided as a supplementary Research Element. Two-sided P values < .05 were considered statistically significant.

## 3 Results

### 3.1 Sample characteristics

Among the 106,005 adolescents included in the analyses, 32.2% reported not watching Mukbang or Cookbang, 30.3% watched ≤3 times/month, 26.4% watched 1–4 times/week, and 11.1% watched ≥5 times/week; two-thirds (67.8%) had viewed such content at least occasionally during the preceding 12 months, and 37.5% viewed it at least weekly. Non-viewers, who formed the largest single category (n = 33,491), differed systematically from frequent viewers: they were more often male (66.3% vs 38.1%) and less likely to report ever alcohol use (27.9% vs 38.3%), ever smoking (7.3% vs 10.8%), or depressive symptoms (23.8% vs 35.1%). The proportion reporting toothache rose monotonically across viewing categories, from 18.4% among non-viewers to 24.8% among those watching ≥5 times/week, as did the proportion reporting gingival pain/bleeding (15.8% to 21.1%; all P < .001) (Table 1).

### 3.2 Dietary behaviours across viewing categories

Late-night snacking and fast-food consumption increased monotonically with viewing frequency (from 1.34 to 1.67 days/week and from 1.85 to 2.39 occasions/week respectively; both P < .001). Unadjusted sugary beverage consumption was not monotonic: 3.84 occasions/week among non-viewers, 3.69 among those watching ≤3 times/month, 3.96 among weekly viewers, and 4.61 among those watching ≥5 times/week. After adjustment for the covariates of the main model, adolescents watching ≥5 times/week consumed .82 (95% CI, .72–.91) more sugary drinks per week, ate late-night snacks on .30 (.25–.34) more days per week, and consumed fast food .54 (.48–.60) more times per week than non-viewers (Table 1).

### 3.3 Association of mukbang/Cookbang viewing with oral symptoms

In design-based logistic regression models adjusting for all covariates, Mukbang/Cookbang viewing frequency was independently associated with both oral symptoms in a dose-response pattern (Table 2). Compared with non-viewers, the adjusted odds of toothache were 1.12 (95% CI, 1.07–1.17) for those watching ≤3 times/month, 1.14 (1.09–1.19) for 1–4 times/week, and 1.21 (1.14–1.28) for ≥5 times/week (P for trend < .001). The corresponding aORs for gingival pain/bleeding were 1.13 (1.08–1.18), 1.16 (1.11–1.21), and 1.22 (1.15–1.30) (P for trend < .001).

### 3.4 Decomposition through dietary behaviours

For the contrast between viewing ≥5 times/week and not watching, the design-based total effect on toothache was an odds ratio of 1.20 (95% CI, 1.14–1.27), of which the natural indirect effect operating jointly through the three dietary behaviours was 1.056 (1.049–1.064) and the natural direct effect 1.14 (1.08–1.20), corresponding to a proportion mediated of 29.6% (22.2–42.7). For gingival pain/bleeding the total effect was 1.22 (1.15–1.28), the natural indirect effect 1.036 (1.030–1.042), and the proportion mediated 18.0% (13.2–25.5) (Table 3).

Sugary beverage consumption and late-night snacking each carried a significant indirect effect, whereas fast-food consumption did not (toothache 1.004, 95% CI .999–1.008; gingival pain/bleeding 1.001, .996–1.005). The relative magnitude of the two contributing mediators was not stable to the scaling of the mediator items: sugary beverages carried the larger indirect effect when ordered category scores were used, whereas the two were of comparable magnitude, or late-night snacking marginally the larger, when the items were rescaled to their approximate weekly frequencies (Supplementary Table S3). Both pathways contributed, but their relative magnitude was sensitive to mediator scaling, so we do not privilege one over the other.

### 3.5 Social patterning and sensitivity analyses

For toothache, the association was modified by sex (P for interaction < .001) and school level (P = .019). It was stronger among females (aOR 1.35; 95% CI, 1.25–1.47) than among males (1.05; .95–1.16), and among middle-school students (1.32; 1.21–1.44) than among high-school students (1.14; 1.05–1.24). Consistent with the latter, entering age as a continuous variable showed the association weakening with increasing age (ratio of odds ratios .988 per year; .979–.998; P = .019). No modification was observed by depressive symptoms (P = .091) or household economic status (P = .664). For gingival pain/bleeding, the association was significant and of similar magnitude in every subgroup examined, with no evidence of effect modification (all P > .05) (Fig. 1; Supplementary Table S4).

Successive adjustment for smartphone use time, sleep duration, sedentary time, perceived stress, dental-service use, and academic performance attenuated the ≥5 times/week association only modestly: for toothache from 1.21 (1.14–1.28) to 1.16 (1.09–1.24), and for gingival pain/bleeding from 1.22 (1.15–1.30) to 1.20 (1.12–1.28). Refitting the main model within the restricted complete-case sample used for the fully adjusted model (n = 94,964) gave 1.19 and 1.21 respectively, indicating that most of this attenuation reflected sample restriction rather than covariate adjustment (Supplementary Table S1). The decomposition was similarly stable across specifications: the proportion mediated ranged from 24.4% to 31.8% for toothache and from 12.4% to 19.0% for gingival pain/bleeding under a model allowing exposure–mediator interaction, under the extended covariate set, and under two alternative mediator scalings, in every case falling within the confidence interval of the primary estimate (Supplementary Table S2).

## 4 Discussion

In this nationally representative sample of more than 100,000 Korean adolescents, more frequent viewing of Mukbang and Cookbang was associated, in a dose-response manner, with higher odds of both toothache and gingival pain/bleeding, independent of oral-hygiene behaviours and other potential confounders. A design-based decomposition indicated that sugary beverage intake and late-night snacking, but not fast-food intake, carried statistically significant indirect effects consistent with the hypothesised dietary pathway, together accounting for 29.6% of the total association with toothache and 18.0% of that with gingival symptoms; because the data are cross-sectional, these proportions describe a statistical decomposition rather than a verified mechanism. Pre-specified subgroup analyses indicated that the association with toothache was more pronounced among females and among middle-school students, whereas the association with gingival pain/bleeding was uniform across subgroups. To the author's knowledge, this is the first nationally representative study to position digital food-environment exposure as a population-level correlate of adolescent oral symptoms.

A growing body of research using Korean adolescent data has documented that Mukbang and Cookbang viewing is associated with unhealthy dietary behaviours (Kim & Oh, 2024; Sung et al., 2024), higher BMI status (Park et al., 2024), content features such as overeating and provocative themes (Kang et al., 2020), and a measurable subset of problematic viewing patterns (Kircaburun et al., 2021). The present findings extend this literature by showing that these behavioural correlates are accompanied by measurable differences in patient-reported oral symptoms at population scale. Mechanistically, the immersive, narrative-rich video format of digital eating-broadcast content engages reward circuitry more potently than static food advertising in paediatric neuroimaging studies (Yeum et al., 2023), providing biological plausibility for stronger downstream dietary effects than would be expected from earlier-generation food media. The substantial direct effect remaining after accounting for the three dietary behaviours indicates that other pathways may also be involved: cariogenic snacking outside structured meals, cue-induced eating in the absence of physiological hunger, or shifts in salivary protective factors accompanying frequent acid challenges. Late-night sugar exposures are of particular interest because nocturnal reductions in salivary flow prolong oral sugar clearance and sustain low plaque pH; observational evidence in children and adolescents indicates that eveningness-associated lifestyle patterns, namely late bedtimes, irregular meal timing, and frequent snacking, are associated with higher caries prevalence (Nishide et al., 2019).

Our data did not support an indirect pathway through fast-food intake, but this is not interpreted as evidence that fast food is of low cariogenic potential. Fast-food meals are habitually consumed together with sugary drinks and sweet desserts, and their refined-carbohydrate content is itself fermentable; the three mediators examined here are positively correlated, and the fast-food contribution is estimated conditional on the other two, so any shared component of the pathway will be attributed to the beverage and snacking terms. Moreover, what governs caries risk is the frequency and clearance of sugar exposure rather than the number of eating occasions of any particular food, and a 7-day frequency measure of fast-food consumption captures this only indirectly (Moynihan & Kelly, 2014; Valenzuela et al., 2021). The null pathway is therefore best regarded as unresolved rather than as informative about the cariogenicity of fast food.

### 4.1 Population-health interpretation and policy implications

Although the observed individual-level associations were modest (aORs of approximately 1.2 at the highest exposure level), three population-health considerations justify a substantive public-health response. First, exposure prevalence is high: two-thirds of Korean adolescents view Mukbang or Cookbang content at least occasionally, more than a third view it weekly or more often, and one in nine views it near-daily. This combination of modest individual effects and widespread exposure is the canonical configuration in which population-level prevention, rather than high-risk intervention, is the more efficient strategy (Rose, 1985). Second, the dietary behaviours implicated in the decomposition are themselves established population-level drivers of caries and of broader cardiometabolic risk, so policy actions that reduce these exposures are likely to yield co-benefits beyond oral health (Peres et al., 2019; Swinburn et al., 2019). Third, the relevant exposure is concentrated during a developmental window in which dietary and oral-health behaviours consolidate into lifelong patterns.

Read through a commercial-determinants-of-health lens (Maani et al., 2020), these findings bear on a policy architecture that already exists in South Korea but does not reach the exposure measured here. The Special Act on the Safety Management of Children's Dietary Life (Ministry of Government Legislation, 2024) covers all students through high school, and the “children's preferred foods” enumerated in its Enforcement Decree, which include confectionery, sweetened and carbonated beverages, instant noodles, hamburgers, pizza, and fried street foods, correspond closely to the fare of eating-broadcast content. Neither the protected class nor the food categories, then, would need to be created; what is missing is the medium. Like the broadcast-advertising frameworks that have historically governed food marketing to children (Boyland et al., 2022; Kelly et al., 2010), the advertising restriction under Article 10 (2) reaches only children's-target programming on television or real-time internet multimedia broadcasting, and its duty-bearers are food business operators rather than platforms or content creators, so user-generated video falls outside it altogether. The most recent amendment, effective February 2026, is instructive in its direction: it widened the range of media while narrowing programme coverage, so that Korean regulation has contracted in scope precisely as adolescent exposure has migrated to channels it does not cover.

Korea has, moreover, already attempted to address this content directly. The 2018 National Obesity Management Plan, issued jointly by nine ministries, announced a guideline and monitoring system for “binge-eating-promoting media”, explicitly naming television and internet broadcasting (Ministry of Health and Welfare, 2018); the proposal met sustained public opposition on freedom-of-expression grounds and was ultimately recast as voluntary recommendation. That debate proceeded almost entirely without population-level evidence on health outcomes, the deficiency to which the present findings speak, and it is a reminder that content-directed regulation of eating broadcasts is politically contested in a way that advertising regulation is not. Against this background, extending the media scope of Article 10 (2) is the most conservative of the levers our findings suggest, since the protected class, the food categories, and the enforcement machinery all already exist. Disclosure of creator–brand relationships operates under a separate instrument, the Korea Fair Trade Commission's guidelines on endorsements and testimonials (Korea Fair Trade Commission, 2024), but reaches advertisers rather than creators; school-based media literacy confronts a statutory gap, the Special Act obliging only elementary schools to provide regular dietary education; and modification of recommendation algorithms, the most consequential lever, is by a considerable margin the least tractable. We advance these as hypotheses for intervention and natural-experiment research rather than as conclusions our design can license. Clinically, dentists and paediatricians counselling adolescents may find it useful to ask about food-media viewing as part of routine dietary-behavior assessment, particularly in younger adolescents and in female patients, in whom the association was strongest.

### 4.2 Social patterning and health-equity considerations

The subgroup findings for toothache warrant comment, and they turn on a single distinction. Toothache is a pain report, whereas gingival bleeding is closer to an observed sign, and the two also differ in latency: gingival inflammation responds within days to plaque accumulation and to the frequency of fermentable-carbohydrate exposure, whereas toothache generally signals a carious lesion that has reached dentine, an endpoint that integrates cariogenic exposure over months to years and is correspondingly more filtered through cumulative individual susceptibility. Both properties predict what was observed: a short-latency, frequency-driven sign responds comparatively uniformly to a behavioural exposure, whereas a longer-latency, self-reported symptom displays heterogeneity, and that heterogeneity should track differences in reporting threshold. This accounts for the stronger toothache association in females and its absence in males (Lukacs, 2011; Sung et al., 2024), for the uniformity of the gingival association across every stratum examined, and for the greater toothache association among middle-school students, in whom eruption of the second permanent molars and the extended period of post-eruptive enamel maturation leave occlusal surfaces unusually caries-susceptible (Steinberg, 2008). Sex composition also explains the non-monotonic crude gradient in sugary beverage intake: non-viewers were predominantly male, and male adolescents consume more sugary drinks, so the gradient emerges only after adjustment. These interpretations are hypotheses suggested by the data rather than propositions tested within it.

Effect modification by depressive symptoms was an exploratory hypothesis, motivated by the documented links between depression and poorer oral health through reduced self-care, altered dietary patterns, and bidirectional inflammatory mechanisms (Joury et al., 2023; Kalaigian & Chaffee, 2023). No evidence of it was found. The association with toothache was somewhat closer to the null among adolescents reporting depressive symptoms, but the difference was compatible with chance and the estimate in that subgroup remained above unity; it therefore provides no basis for regarding digital food-environment exposure as benign, still less protective, in this group. The null result for household economic status should likewise not be read as evidence that the association is socially uniform. Household economic status is measured in the KYRBS by adolescent self-report, and adolescents report family economic position with limited accuracy; non-differential misclassification of a modifier biases an interaction test towards the null. Whether the digital food environment cuts across or reinforces the well-documented socioeconomic gradients in oral disease (Peres et al., 2019) therefore remains open.

### 4.3 Strengths and limitations

Strengths include the very large, nationally representative sample drawn from a probability-based national surveillance system; design-based analyses that account for survey stratification, clustering, and weighting throughout, including in the mediation analysis and its bootstrap; a counterfactual decomposition that does not rely on the rare-outcome approximation; and pre-specified subgroup tests with explicit a priori hypotheses. Several limitations warrant explicit comment.

First, the cross-sectional design precludes causal inference. The mediation estimates reported here are a design-based statistical decomposition of associations observed at a single point in time; although the estimands carry the formal names of natural direct and natural indirect effects, a causal reading would require sequential ignorability and a verified exposure→mediator→outcome temporal ordering, neither of which a cross-sectional survey can supply. The temporal structure of the KYRBS items compounds this concern: viewing and oral symptoms both refer to the preceding 12 months, whereas the dietary mediators refer to the preceding 7 days, so the measurement windows do not instantiate the assumed causal sequence. Reverse causation cannot be excluded, and it may operate at more than one point in the pathway: oral discomfort could alter viewing behavior, and, more plausibly, adolescents with an established preference for sweet, energy-dense foods may be more drawn to eating-broadcast content, with recommendation algorithms then reinforcing that exposure. Under such a mechanism the dietary behaviours treated here as mediators would be partly antecedent to the exposure, and the decomposition would misattribute a shared source of variation to an indirect pathway. Cross-sectional mediation proportions are also unstable, as the width of the design-based confidence intervals makes plain (22.2–42.7% for toothache; 13.2–25.5% for gingival symptoms). Longitudinal data with mediators measured between exposure and outcome are required to establish directionality and to provide dependable estimates of mediated proportions.

Second, both outcomes are patient-reported rather than clinically examined. Single-item symptom measures of this kind are standard in national oral-health surveillance and capture the functionally important dimension of disease, namely pain that interferes with eating, sleeping, and school attendance (Petersen, 2008), but their limitations are substantial. The items have not been validated against clinical examination in Korean adolescents, so their sensitivity and specificity for dentine caries and gingivitis are unknown. A single toothache item aggregates aetiologically distinct conditions, including caries, pulpitis, dentine hypersensitivity, and pericoronitis around erupting third molars. Symptom reporting also depends on pain thresholds, on health literacy, and on contact with dental services, and unmet dental care need is consistently more prevalent in Korea than unmet medical care need (Kim et al., 2024); dental-service use in the preceding 12 months was adjusted for in a sensitivity analysis with little change in the estimates (Supplementary Table S1), but a dental visit is an imperfect proxy for unmet need, which the KYRBS does not measure. Finally, the argument that misclassification is likely non-differential with respect to viewing frequency, and that the estimates are consequently conservative, is an assumption that cannot be tested in these data.

Third, the exposure is measured by a single item that combines eating-broadcast and cooking-broadcast content, which the KYRBS does not distinguish. The two plausibly differ in portrayed portion size, eating pace, and commercial intent, and a combined measure will attenuate any content-specific effect. Content-level features (specific foods featured, host eating speed, portion sizes), viewing platform, device ownership, and parental restrictions on device use were likewise unavailable and would help refine mechanistic interpretation.

Fourth, residual confounding cannot be ruled out. The attenuation under the extended covariate set was slight (Section 3.5), which argues against the association being merely a proxy for heavy screen use, but the KYRBS measures smartphone use time rather than total screen time across devices and includes no smartphone-dependence scale, so heavy use cannot be distinguished from problematic use; nor does it measure family or individual health consciousness, for which only oral-hygiene behavior and academic performance serve as partial proxies.

Finally, the pooled data span 2022 and 2025 and therefore straddle a period of documented change in adolescent oral-hygiene behavior (Oh et al., 2023). Oral-hygiene behaviours were adjusted for throughout, and survey year is absorbed within the pooled design, but secular change in the exposure or in symptom reporting cannot be excluded.

### 4.4 Conclusion

In a nationally representative sample of more than 100,000 Korean adolescents, more frequent exposure to digital eating-broadcast (Mukbang) and cooking-broadcast (Cookbang) media was associated with dose-dependent increases in patient-reported toothache and gingival pain/bleeding, with a substantial part of the association attributable, in a cross-sectional decomposition, to sugary beverage intake and late-night snacking. Read through population-health and commercial-determinants-of-health lenses, these findings identify the digital food environment as a candidate structural target for adolescent oral-health policy, and supply the population-level evidence whose absence has hitherto constrained policy debate in this area. They do not, on their own, establish that reducing exposure would reduce oral symptoms. Longitudinal studies linking specific content features to clinically examined oral-health endpoints, and intervention or natural-experiment designs capable of testing the policy levers discussed here, are now needed. Surveys should also separate eating-broadcast from cooking-broadcast content, which the present exposure measure could not distinguish.

## Ethical approval

The Korea Youth Risk Behavior Web-based Survey protocol was approved by the Korea Disease Control and Prevention Agency. The present secondary analysis of de-identified, publicly released data was exempted from review by the Institutional Review Board of Dankook University (reference number: DKU IRB 2026-02-004).

## Data and code availability

The KYRBS data are publicly available from the Korea Disease Control and Prevention Agency (https://www.kdca.go.kr/yhs/) upon request and after approval of a use proposal. Analysis code is available from the author on reasonable request.

## Ethical statement

This study analysed de-identified, publicly available secondary data from the Korea Youth Risk Behavior Web-based Survey (KYRBS), an anonymous nationwide health-behavior surveillance system conducted by the Korea Disease Control and Prevention Agency (KDCA). The KYRBS protocol was approved by the KDCA, which obtained electronic informed assent from all participating adolescents prior to survey administration. All data are released to researchers in fully de-identified form after approval of a data-use proposal.

Because the present analysis was conducted exclusively on de-identified, publicly released secondary data and did not involve any direct contact with human participants, the study was reviewed and granted exemption from full ethical review by the Institutional Review Board of Dankook University (reference number: DKU**-**2026-02-004). The basis for exemption was the use of anonymised, publicly available data with no identifiers that could be linked to individual participants.

All applicable national and institutional regulations governing research involving human-subject data were followed. The privacy rights of participants were observed throughout the conduct of this research, and no attempt was made to re-identify any individual respondent.

This research was conducted in accordance with the principles of the Declaration of Helsinki and complies with the International Committee of Medical Journal Editors (ICMJE) recommendations for the conduct, reporting, editing, and publication of scholarly work in medical journals.

## Declaration of generative AI and AI-assisted technologies in the writing process

During the preparation of this work, the author used generative AI tools in order to improve language clarity and manuscript organization. After using these tools, the author reviewed and edited the content as needed and takes full responsibility for the content of the publication.

## Funding

The present research was supported by the research fund of 10.13039/501100002467Dankook University in 2026. The funder had no role in study design, data collection or analysis, manuscript preparation, or the decision to submit for publication.

## Declaration of competing interests

The authors declare that they have no known competing financial interests or personal relationships that could have appeared to influence the work reported in this paper.

## Data Availability

KYRBS data are publicly available from the Korea Disease Control and Prevention Agency (https://www.kdca.go.kr/yhs/) upon approval of a data-use proposal.
