# Supplementary material for: Mukbang/Cookbang viewing and adolescent oral symptoms: A nationally representative analysis of social patterning in South Korea
Source: SSM Popul Health. 2026 Aug 9;35:101959. doi: 10.1016/j.ssmph.2026.101959 (PMC13486880; doi:10.1016/j.ssmph.2026.101959)
Supplement: Supplementary file 1 [file mmc1.docx]

**SUPPLEMENTARY MATERIAL**

**Supplementary Table S1. Sensitivity of the main association to additional confounders**

| **Model** | **≤3/month** | **1–4/week** | **≥5/week** | ***n*** |
| --- | --- | --- | --- | --- |
| **Toothache** |  |  |  |  |
| M1 Main model (primary) | 1.12 (1.07–1.17) | 1.14 (1.09–1.19) | 1.21 (1.14–1.28) | 106,005 |
| M2 M1 + smartphone use time | 1.11 (1.06–1.16) | 1.13 (1.08–1.18) | 1.18 (1.11–1.26) | 106,005 |
| M3 M2 + sleep duration, sedentary time, perceived stress | 1.12 (1.07–1.18) | 1.12 (1.07–1.17) | 1.15 (1.08–1.23) | 94,964 |
| M4 M3 + dental-service use, academic performance | 1.12 (1.07–1.17) | 1.12 (1.07–1.18) | 1.16 (1.09–1.24) | 94,964 |
| M1b Main model, restricted to the M4 complete-case sample | 1.12 (1.07–1.18) | 1.12 (1.07–1.18) | 1.19 (1.12–1.27) | 94,964 |
| **Gingival pain/bleeding** |  |  |  |  |
| M1 Main model (primary) | 1.13 (1.08–1.18) | 1.16 (1.11–1.21) | 1.22 (1.15–1.30) | 106,005 |
| M2 M1 + smartphone use time | 1.13 (1.08–1.18) | 1.15 (1.10–1.21) | 1.21 (1.14–1.28) | 106,005 |
| M3 M2 + sleep duration, sedentary time, perceived stress | 1.13 (1.07–1.18) | 1.17 (1.12–1.23) | 1.19 (1.11–1.26) | 94,964 |
| M4 M3 + dental-service use, academic performance | 1.12 (1.07–1.18) | 1.17 (1.12–1.23) | 1.20 (1.12–1.28) | 94,964 |
| M1b Main model, restricted to the M4 complete-case sample | 1.12 (1.07–1.18) | 1.17 (1.12–1.23) | 1.21 (1.14–1.29) | 94,964 |

Values are adjusted odds ratios (95% CI) relative to non-viewers, from design-based logistic regression. All models include the covariates of the main model. Smartphone use time is the weighted average of self-reported weekday and weekend use (hours/day). Sedentary time is the weighted average of weekday and weekend sitting time. Model M1b repeats the main model in the reduced complete-case sample of model M4, isolating attenuation attributable to covariate adjustment from that attributable to sample restriction.

**Supplementary Table S2. Sensitivity of the mediation analysis**

| **Model** | **Total effect** | **Natural direct effect** | **Natural indirect effect** | **Proportion mediated, %** |
| --- | --- | --- | --- | --- |
| **Toothache** |  |  |  |  |
| M1 Primary | 1.20 (1.14–1.27) | 1.14 (1.08–1.20) | 1.056 (1.049–1.064) | 29.6 (22.2–42.7) |
| M2 With exposure × mediator interaction | 1.20 (1.14–1.28) | 1.15 (1.08–1.22) | 1.048 (1.038–1.059) | 25.4 (18.2–39.1) |
| M3 Extended confounder set (*n* = 94,964) | 1.16 (1.09–1.22) | 1.11 (1.05–1.17) | 1.039 (1.033–1.045) | 26.4 (19.1–46.5) |
| M4 Mediators rescaled to weekly frequency | 1.20 (1.14–1.28) | 1.15 (1.09–1.22) | 1.046 (1.038–1.053) | 24.4 (17.7–35.1) |
| M5 Mediators log-transformed | 1.20 (1.14–1.28) | 1.13 (1.07–1.20) | 1.061 (1.053–1.069) | 31.8 (22.9–45.5) |
| **Gingival pain/bleeding** |  |  |  |  |
| M1 Primary | 1.22 (1.15–1.28) | 1.17 (1.11–1.24) | 1.036 (1.030–1.042) | 18.0 (13.2–25.5) |
| M2 With exposure × mediator interaction | 1.21 (1.16–1.28) | 1.17 (1.11–1.23) | 1.036 (1.026–1.045) | 18.3 (12.1–28.0) |
| M3 Extended confounder set (*n* = 94,964) | 1.19 (1.12–1.26) | 1.17 (1.10–1.23) | 1.022 (1.016–1.029) | 12.4 (8.3–21.1) |
| M4 Mediators rescaled to weekly frequency | 1.22 (1.15–1.29) | 1.18 (1.12–1.25) | 1.030 (1.024–1.035) | 15.0 (10.7–21.4) |
| M5 Mediators log-transformed | 1.22 (1.16–1.29) | 1.17 (1.11–1.24) | 1.038 (1.032–1.044) | 19.0 (13.9–27.0) |

Values are odds ratios (95% CI) for the contrast ≥5 times/week versus not watching, estimated as in Table 3. Model M2 allows the effect of each mediator on the outcome to vary with viewing frequency. Model M3 additionally adjusts for smartphone use time, sleep duration, sedentary time, perceived stress, dental-service use, and academic performance. Models M4 and M5 vary the scaling of the mediators: M4 replaces the ordered category score with the approximate weekly consumption frequency (category midpoints), and M5 applies a logarithmic transformation to that frequency. The total effect is invariant to mediator scaling, as expected. The proportion mediated ranged from 24.4% to 31.8% for toothache and from 12.4% to 19.0% for gingival pain/bleeding across all specifications, in every case within the bootstrap confidence interval of the primary estimate. Confidence intervals are from 250 stratified bootstrap resamples of primary sampling units (2,000 for model M1).

**Supplementary Table S3. Mediator-specific indirect effects under alternative mediator scalings**

| **Mediator scaling** | **Via sugary beverages** | **Via late-night snacking** | **Via fast food** | **PM, %** |
| --- | --- | --- | --- | --- |
| **Toothache** |  |  |  |  |
| Ordered category score (primary) | 1.030 (1.025–1.035) | 1.022 (1.018–1.026) | 1.004 (0.999–1.008) | 29.6 |
| Approximate weekly frequency | 1.022 (1.019–1.027) | 1.023 (1.019–1.028) | 1.000 (0.995–1.004) | 24.4 |
| Log-transformed frequency | 1.030 (1.025–1.036) | 1.023 (1.019–1.028) | 1.006 (1.001–1.011) | 31.8 |
| **Gingival pain/bleeding** |  |  |  |  |
| Ordered category score (primary) | 1.019 (1.015–1.024) | 1.015 (1.012–1.019) | 1.001 (0.996–1.005) | 18.0 |
| Approximate weekly frequency | 1.014 (1.010–1.018) | 1.016 (1.013–1.020) | 0.999 (0.995–1.003) | 15.0 |
| Log-transformed frequency | 1.019 (1.015–1.023) | 1.016 (1.012–1.020) | 1.002 (0.998–1.007) | 19.0 |

Values are interventional indirect effect odds ratios (95% CI) for the contrast ≥5 times/week versus not watching. PM, proportion mediated by all three mediators jointly. Sugary beverage consumption and late-night snacking carried significant indirect effects under every scaling, whereas fast-food consumption did not do so consistently. The relative magnitude of the two leading mediators was not stable to mediator scaling: sugary beverages carried the larger indirect effect when ordered category scores were used, whereas the two were of comparable magnitude, or late-night snacking marginally the larger, when the items were rescaled to their approximate weekly frequencies. Both pathways contributed, but their relative magnitude was sensitive to mediator scaling, so no priority is assigned to either.

**Supplementary Table S4. Exposure × mediator interaction and effect modification by age**

| **Term** | **Toothache** | **Gingival pain/bleeding** |
| --- | --- | --- |
| **Exposure × mediator interaction, β (*P*)** |  |  |
| Viewing × sugary beverage intake | −0.0157 (*P* = .011) | +0.0126 (*P* = .057) |
| Viewing × late-night snacking | −0.0032 (*P* = .507) | −0.0022 (*P* = .675) |
| Viewing × fast-food intake | +0.0045 (*P* = .634) | −0.0144 (*P* = .146) |
| **Effect modification by age (continuous, 12–18 y)** |  |  |
| Viewing × age, ratio of ORs per year of age | 0.988 (0.979–0.998); *P* = .019 | 0.996 (0.986–1.006); *P* = .403 |

β values are coefficients on the log-odds scale from design-based logistic regression, with Mukbang/Cookbang viewing entered as an ordinal variable (0–3) and the mediators as ordered category scores. The age model replaces the grade indicator variables with continuous age (centred at 15 years). All models are adjusted for the covariates of the main model.
